# Magnetic Angular Rate and Gravity Sensor Based Supervised Learning for Positioning Tasks

**DOI:** 10.3390/s19245364

**Published:** 2019-12-05

**Authors:** Balázs Nagy, János Botzheim, Péter Korondi

**Affiliations:** Department of Mechatronics, Optics and Mechanical Engineering Informatics, Faculty of Mechanical Engineering, Budapest University of Technology and Economics, 4-6 Bertalan Lajos Street, 1111 Budapest, Hungary; botzheim@mogi.bme.hu (J.B.); korondi@mogi.bme.hu (P.K.)

**Keywords:** MARG sensor, supervised learning, sensor fusion, position estimation

## Abstract

This paper deals with sensor fusion of magnetic, angular rate and gravity sensor (MARG). The main contribution of this paper is the sensor fusion performed by supervised learning, which means parallel processing of the different kinds of measured data and estimating the position in periodic and non-periodic cases. During the learning phase, the position estimated by sensor fusion is compared with position data of a motion capture system. The main challenge is avoiding the error caused by the implicit integral calculation of MARG. There are several filter based signal processing methods for disturbance and noise estimation, which are calculated for each sensor separately. These classical methods can be used for disturbance and noise reduction and extracting hidden information from it as well. This paper examines the different types of noises and proposes a machine learning-based method for calculation of position and orientation directly from nine separate sensors. This method includes the disturbance and noise reduction in addition to sensor fusion. The proposed method was validated by experiments which provided promising results on periodic and translational motion as well.

## 1. Introduction

Estimation of the orientation and position of a robot is essential for robot navigation. The navigation system is an important part of an autonomous mobile robot. There are numerous practical applications which use global positioning system (GPS) [1,2], onboard camera [3,4], light detection and ranging (LIDAR) sensor [5,6] or other external observation system [7,8].

Navigating a robot requires a reliable solution that can be implemented even on robots with limited computational capacity. The concept of iSpace is based on the idea that computationally expensive algorithms can be outsourced from the onboard computer of the robot into the environment of the robot [9]. In this setup, there is no need for a powerful computer with high computation capacity onboard [10]. In this case, the energy consumption can be maintained at a lower level on the mobile robot so the battery life increases. However, the previously mentioned applications have some major disadvantages. External sensors with wireless connections may lose connection with the moving agent causing navigation breakdown in this way. These applications are sensitive to the environment. On the other hand image processing or 3D point, cloud-based applications have a high demand for computational capacity to perform simultaneous localization and mapping (SLAM) [11]. These methods are not suitable for small robots with limited computational capacity. Generally speaking, most of the localization algorithms use a predefined map. Building a map from the beginning is a challenging task. A fully automated small robot has to be able to run independently from external sensors, using only onboard sensors. In this manner, a magnetic, angular rate and gravity sensor (MARG) sensor seems to be a good choice to be a sensor.

The main interest of Madgwick et al. was to implement an algorithm, which can calculate the distance information from a MARG sensor. In this application [12,13] the MARG sensor was mounted on a leg of a human being and an Arduino collected the data. After the measurement, the algorithm calculated the path of the sensor. The calculation was performed offline, as opposed to real-time, and the unique features of the test case were programmed into the algorithm. The calculation was based on the stepping frequency of the walking motion. Frequency analysis can provide useful information only in the case of periodic movements. During walking another notable feature is that the leg hits the floor in every step. This impact is detectable and can be used to eliminate the collected error during one step. This correction can prevent the cumulated error in position data. Since the measured signal is acceleration the algorithm has to perform a double integral step to estimate the position. Integrating noise is the main problem because noise integration is the main cause of the shifting position signal. The shifting error can be compensated by additional sensor fusion [14]. The main question is whether a similar algorithm can be extended to deal with non-periodic movement in a more general case. This question is also investigated in this paper.

It is proven that MARG sensors are capable of estimating the orientation of the sensors [15]. The accelerometer provides information about the gravitational force, the gyroscope can handle the quick movement and the magnetometer can prevent the long term shifting error. The fusion of these three sensors for orientation estimation tasks combines the advantages of each sensor. The complementary filter [16] is a fast and computationally inexpensive method to estimate orientation from MARG sensor data.

The estimation of the position is more challenging because of the sensory noise and disturbance. Several methods are introduced for disturbance rejection in the frequency domain, an overview is published in [17]. This paper is an application-oriented paper, where machine learning can be applied for sensor fusion and disturbance rejection. Machine learning techniques are very popular for handling complex problems and providing solutions for regression, classification, and optimization tasks [18,19]. In this paper deep learning [20] is applied using a long short-term memory (LSTM) based neural network architecture [21].

Recently a popular topic is human activity recognition, where deep learning algorithms are used to classify human motions [22,23,24]. These applications use the data from a smartphone accelerometer. Smartphones are powerful computers to run deep learning networks, but these applications provide solutions for a classification problem. Predicting velocity or position is a regression problem. A smartphone sensor-based walking speed estimation algorithm is developed by Shrestha et al. [25]. This paper proposes a regression method with deep learning to predict speed. Position estimation is a complex task even with using neural networks. Kalman filter is developed to address this task [26]. A pre-defined model is used to reduce noise and predict the position from accelerometer data. The algorithm cumulates errors during the run but works well in a short timeperiod. According to the measurements, this algorithm worked for approximately 10 s. After this time the prediction becomes untrustworthy.

The structure of the paper is as follows. In Section 2 the problem statement is described. Section 3 details the proposed methodology. In Section 4 simulation results and measurement results are presented and discussed. Section 5 concludes the paper.

## 2. Problem Statement

**Figure 1:**
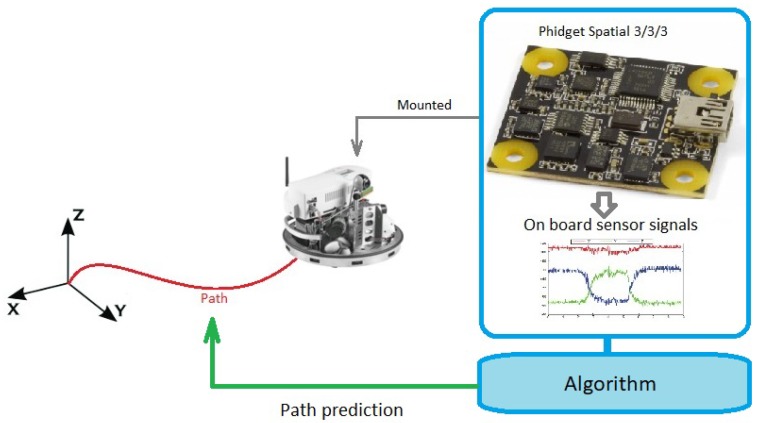
Problem description.

The main goal is to estimate the position of a moving robot or agent by using only sensors mounted on the moving agent. Numerous studies addressed this problem and provided solutions but not every application is suitable for small autonomous robots with limited computational capacity. This study focuses on a possibility to produce a method which does not depend on an external observation system, it uses only onboard sensors and calculations and does not use a wireless connection. The method has to be small enough to mount on a robot that has no capacity to work with image processing or 3D point cloud-based mapping methods. To keep a low computational demand a Phidget Spatial 3/3/3 (Phidgets Inc., Calgary, AB, Canada) [27] magnetic angular rate and gravity sensor was chosen to proceed with the task. The Phidget Spatial 3/3/3 sensor can be seen in Figure 1. The sensor contains a three-axis accelerometer, a three-axis gyroscope, and a three-axis magnetometer.

The study aims to predict position from a sensor fusion using mainly acceleration data. This means that the data has to be integrated. The theoretical relation between acceleration and position is defined by Equation (1).

(1) $$a\left(t\right),\;v\left(t\right)=v_{0}+\int_{0}^{t}a\left(\tau \right)d\tau ,\;r\left(t\right)=r_{0}+\int_{0}^{t}v\left(\tau \right)d\tau ,$$

where *t* is the time, a(t) is the acceleration, v(t) is the velocity and r(t) is the position.

Acceleration data are, however, heavily affected by noise and the integration of measurement noise causes a shift in the position calculation and ruins the prediction. The noise affected relations are defined by Equations (2)–(4).

(2) $$a\left(t\right)+n_{a}\left(t\right)$$

(3) $$v_{0}+\int_{0}^{t}\left(a\left(\tau \right)+n_{a}\left(\tau \right)\right)d\tau =v\left(t\right)+e_{v}\left(t\right)$$

(4) $$r_{0}+\int_{0}^{t}\left(v\left(\tau \right)+e\left(\tau \right)\right)d\tau =r_{0}+\int_{0}^{t}\left(v_{0}+\int_{0}^{\tau_{1}}\left(a\left(\tau_{2}\right)+n_{a}\left(\tau_{2}\right)\right)d\tau_{2}\right)d\tau_{1}=r_{0}+v_{0}t+r\left(t\right)+e_{r}\left(t\right),$$

where *e* is the calculated error and $n_{a}$ is the noise. If the initial conditions are known and $r_{0}=0$ and $v_{0}=0$ then the relation can be reduced to Equation (5).

(5) $$\int_{0}^{t}\int_{0}^{\tau_{1}}\left(a\left(\tau_{2}\right)+n_{a}\left(\tau_{2}\right)\right)d\tau_{2}d\tau_{1}=r\left(t\right)+e_{r}\left(t\right).$$

Double integration of the acceleration data equals the position data but the result of the double integration of the noise is an unwanted shift error ($e_{r}$) in the position estimation. Error handling is a key step but the type of error and the parameters of the error, like the distribution or offset, is not known in every case. In most cases $e_{r}\left(\infty \right)=\infty$. To calculate the position information the necessary information is included in the acceleration measurement but the noise data ratio is high. The position calculation error is also big. In a full case study we have to take into consideration the following disturbance parameters:

- distribution of the noise
- offset of the noise
- occurrence of the noise (sampling time, input feature, reference signal)

These parameters are examined in a simulation or during a real-life test case. Using a MARG sensor to estimate the path of the movement and the current position and orientation of a robot can reduce the calculation needs of a mapping algorithm. Current methods use previously defined maps or image matching or point cloud matching algorithms to build maps. Using pre-defined maps means this map has to be taken by an external observation system and the robot is not independent in this case. Using image or 3D point cloud matching algorithms means, that the robot has to deal with huge databases and perform a lot of computations. If the path of the robot can be estimated by a MARG sensor which produces fewer measurement points means, that the heavy computational load of a SLAM algorithm can be reduced. Instead of simultaneous performance, it can be done in a serial manner. Using the MARG sensor in close or mid-range makes it possible to use the computationally expensive methods on long-range prediction with less frequently. For example, a robot with a blank memory after initialization has to take a picture. During the motion of the robot, pictures have to be taken so frequently that every new picture overlaps with the previous one. In this case, a robot can calculate the distance and build the map simultaneously. With a MARG sensor-based position estimation algorithm the robot has the opportunity to take not only overlapping pictures.

## 3. Proposed Methodology

This study aims a method that has a lower demand for computational capacity, so it can be mounted on small autonomous robots as well. The method is based on an onboard MARG sensor to make the system robust and eliminate the uncertainty of the wireless connections. To achieve this goal our proposed method uses neural networks to process the data provided by a MARG sensor and predict the position and orientation from it.

**Figure 2:**
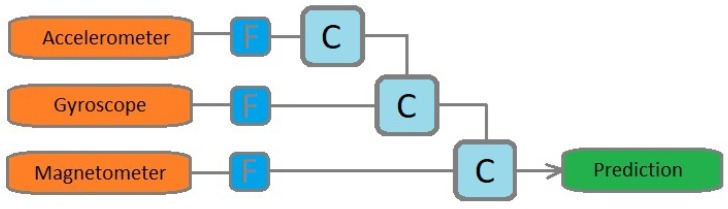
Predefined sequential approach (F: filter; C: calculation)

The solution for the problem can be a neural network which learns how to estimate the robot position according to the sensor data. Existing methods [15,16] approached mainly the orientation estimation task with an explicit model like the schematic illustration in Figure 2. The data of each sensor is filtered separately and after the filtering step, the sensor data are combined according to a predefined order.

**Figure 3:**
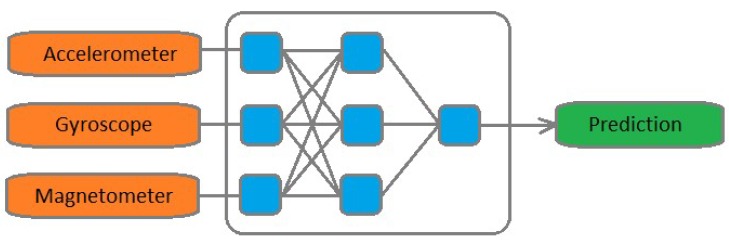
Proposed approach.

The problem with this approach is that the directions and connection point of the sensor fusion are predetermined. The algorithm is not flexible. Using a neural network means, that the algorithm has the flexibility to combine the data of the sensors freely. The proposed architecture can be seen in Figure 3.

**Figure 4:**
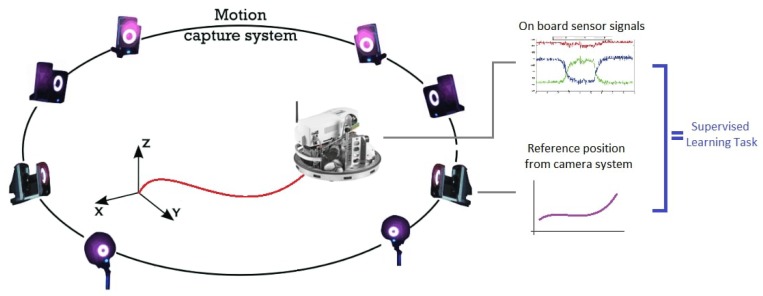
Proposed training system. After the training phase the camera system will not be used anymore.

To teach such a network during the learning phrase an external camera system provides the reference data. In this manner, the task can be interpreted as a supervised learning regression task. Figure 4 shows the schematic drawing of the proposed method.

A synchronised measurement system can log the position of the robot in an absolute coordinate system and the data from the onboard MARG sensor at the same time. Detailed information about the measurement system will be provided in Section 4.4. The external camera system provides the 3 axis position and the orientation of the robot in quaternion form. The onboard MARG sensor provides three-axis acceleration, gyroscope and magnetometer data. The data from the external camera system is used only during the learning phase as reference. After the algorithm learns how to predict the position according to the pattern in the data of the sensor, in the prediction state only the data of the sensor is needed. The implementation on an autonomous mobile robot can be executed according to the following Algorithm 1.

**Algorithm 1** Prediction.
1:**while** Sensor is running **do**2: **if** Data buffer is full **then**3:  Delete the oldest measurement record4: **end if**5: Save new measurement record6: Calculate the time difference from previous measurement7: Run neural network to predict the change in position 8: Calculate the absolute position by summarising the previously calculated position changes9:
**end while**



The aimed solution is mainly based on acceleration data. To estimate position from acceleration data the measurement data has to be integrated. The sensor data are affected by different noises and the integration of noise causes a shift in the position data. Different neural network layer types are capable of filtering or modelling time series data [20]. To examine the effect of different noise types to the learning capability a simulation was executed. The main aim of the simulation is to model some phenomena noticed in real life applications and examine it in a fully controlled environment. The examined test case includes noise on the sampling time, noise on the signal amplitude, and noise on the reference signal.

In the simulation environment a 300 samples wide time window was generated. The sampling time (*T*) was set to a constant value. Additional random noise with Gaussian distribution can modify the sampling time according to Equation (6) to determine the noise affected sampling time (dtime). Using the same noise affected sampling time vector 2 sine functions were generated according to Equations (7) and (8) as input functions. As a desired output, two possible signals were generated using the input signals according to Equations (9) and (10). With the help of these signals the effect of time multiplication and numerical integration can be examined. These type of calculations are likely to be required in a real life scenario.

(6) $$dtime=T+d_{t}$$

where T=0.9 ms and *d* is random noise with Gaussian distribution.

(7) $$A_{1}\cdot sin\left(\omega_{1}\cdot dtime+\varphi_{1}\right)+d_{1}=sin_{1}$$

where $A_{1}=1$, $\omega_{1}=0.5$, $\varphi_{1}=90$ and $d_{1}$ is random noise.

(8) $$A_{2}\cdot sin\left(\omega_{2}\cdot dtime+\varphi_{2}\right)+d_{2}=sin_{2}$$

where $A_{1}=1.5$, $\omega_{1}=0.01$, $\varphi_{1}=0$ and $d_{2}$ is random noise.

(9) $$\left(sin_{1}\cdot dtime+sin_{2}\right)+d_{iY}=iY$$

(10) $$\left(\sum_{k=0}^{t}iY\right)+d_{iY}=ciY_{k}$$

where *t* is the time spent from the start of the measurement.

Universal approximation theorem or universal approximation capability of feedforward neural networks states that any continuous function defined on a closed set can be uniformly approximated to an arbitrary degree of accuracy by a three-layer feedforward neural network [28].

**Figure 5:**
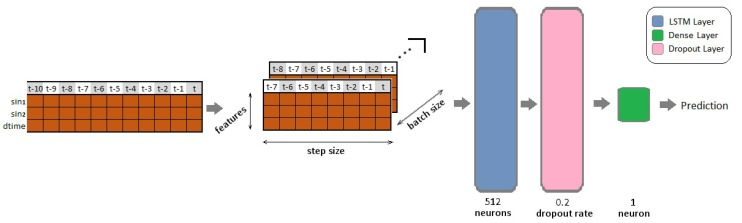
Neural network model used in the simulation.

As a base line solution a simple long short-term memory network was built according to Figure 5. LSTM networks are the best option to fit time series data. The three input parameters are the two sine functions ($sin_{1}$ and $sin_{2}$) and the sampling time (dtime). The desired output functions can be iY or ciY. We used an eight-measurement wide window to take into consideration the previous time steps and set the epoch size to 50. The hyperparameters did not change during the whole study. In this case, we can make a comparison of the layers efficiency in different cases and focus on the effect of the noise. Tests were made using different noise levels. The results are explained in Section 4.2.

**Figure 6:**
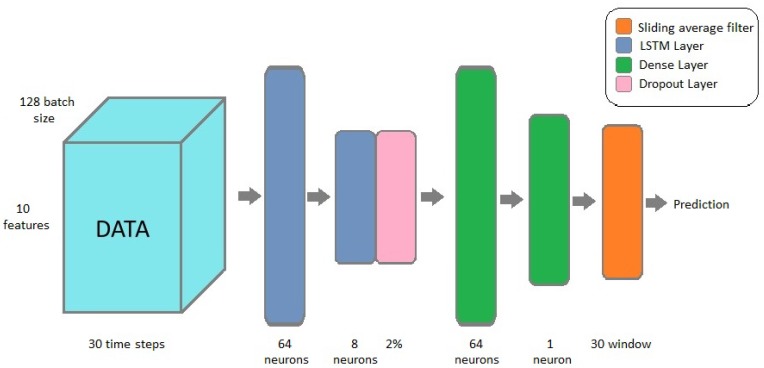
Extended neural network model.

In the second step to improve the performance of the application a deeper neural network was built. The extended neural network architecture is illustrated in Figure 6. The implemented network contains two LSTM layers with 64 and eight neurons, two dense layers with 64 neurons and one neuron, and a sliding average filter is applied at the end of the neural network to make the prediction smoother. The LSTM layers modelled the time series data and the dense layers made the final prediction. To prevent the network from getting stuck in local optimum point a dropout layer is added to the architecture. The result of the extended network on simulated data are explained in details in Section 4.2.

A real test was carried out in order to transfer the knowledge from the simulation case. As a measurement test case, the sensor was mounted on a pendulum. The motion of an ideal pendulum has one degree of freedom. This case is simple enough to test different deep learning algorithms. After testing different algorithms, the method provided acceptable results, the use case can be extended to a more complex motion pattern. The measurements took place in the observation space of a motion capture laboratory containing 18 cameras. The camera system can track the position and orientation of different agents tagged with special markers inside the observation space. The sensor was mounted with three markers to obtain position and orientation. The measurement setup is introduced in detail in Section 4.4.

**Figure 7:**
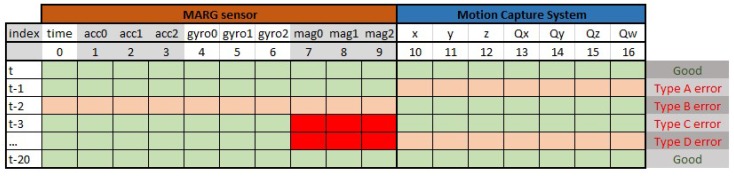
Error types in the measurement.

The position and orientation from the motion capture laboratory serves as a reference, or desired output for a learning task, whilst the sensor data serves as input data for a neural network. The output of the applied neural networks is only one variable in every case. The effect can be examined separately and after the test case, the different output specialized networks can be merged or run in parallel. In these measurements, an additional error type is occurred due to the different sampling frequencies of the different sensors and motion capture laboratory. This error case, shown in Figure 7, is not covered by the simulation environment. In best-case scenario, the MARG sensor data and the motion capture data are synchronized. In some cases, the data are not in align like in the case of type A or type B errors. This can happen when the measurement system sampling time is smaller than the sampling time of the two included systems. These cases can be handled by keeping the data of the motion capture system as long as a new measurement is taken. We wanted to keep every piece of useful information from the MARG sensor and avoiding interpolation for the missing data. Interpolating the missing data would cause the neural network to learn an unwanted calculation inside the data pool. The timing of the MARG sensor is forced in such a way that type B errors do not occur anymore. Type A errors can occur if the motion capture system loses track of the markers, but this case was not present during the measurement. Type C error is the most common error type. Since the magnetometer sensor sampling frequency is less than the sampling frequency of the accelerometer and the gyroscope. Handling type C errors is left to the neural networks. Depending on the measurement setup sampling time option the ratio of the appearance of type C errors can change. Looking at the whole process of learning it is easier to handle this error type inside the neural network. Type D error is a combined case of type A and type C errors, but with the previous ideas, it can be reduced to type C errors.

**Figure 8:**
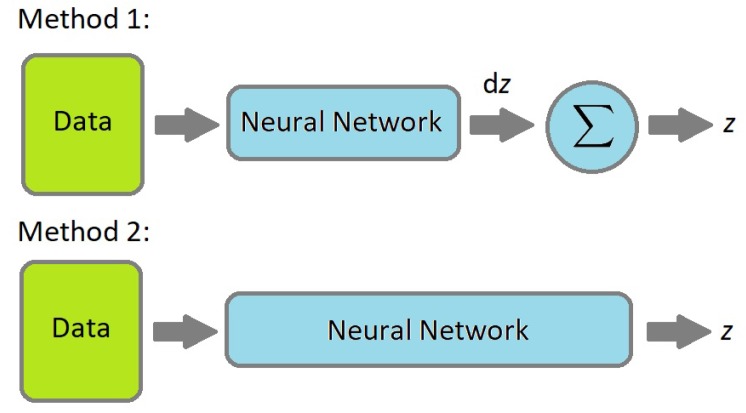
Possible neural network application concepts.

After the error handling a signal preprocessing step was performed. At every time step, a differential position (d*x*, d*y*, d*z*) and time (dtime) are calculated by extracting the new measurement data from the previous step. Neural networks may work better on differential data, because the range is predictable. Thus, two methods are investigated as shown in Figure 8. The application has predictable limits and ranging the data are part of the preprocessing stage as data normalization, also known as feature scaling.

**Figure 9:**
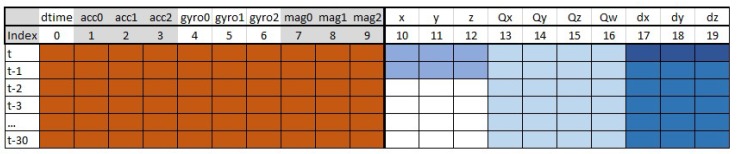
Data frame.

Absolute time stamps (time) from the measurement has to be converted into a ranged signal (dtime). This step can be implemented in the final application as well. The full data frame can be seen in Figure 9. The orange cells serve as the input information to the neural network, while the blue cells are the optional desired outputs of the neural network.

The sliding window size is 30 time step wide. It means LSTM layers can process 30 previous measurements from the past. The used epoch size is 50. The input feature vector contains 10 different features. Using a batch gradient method on one measurement file with 6332 measurements the dimensions of the input tensor is 6324×30×10.

## 4. Experimental Results

To prove the validity of the idea first a simulation was performed. Inside the simulations we tested the effect of different noise types and different neural network layer types on controlled sinusoidal functions. After the simulation a real life test was performed. During the test the MARG sensor was mounted on a pendulum. In this setup the one-degree-of-freedom (DoF) motion of the pendulum was tracked. The source code and the measurement data sets can be seen in the Supplementary Materials. The code development based on the work of Jason Brownlee [29].

### 4.1. Simulation Setup

**Figure 10:**
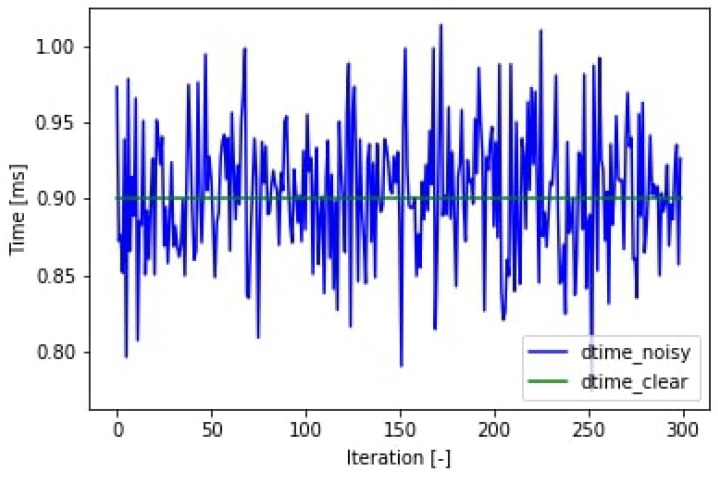
Sampling time.

**Figure 11:**
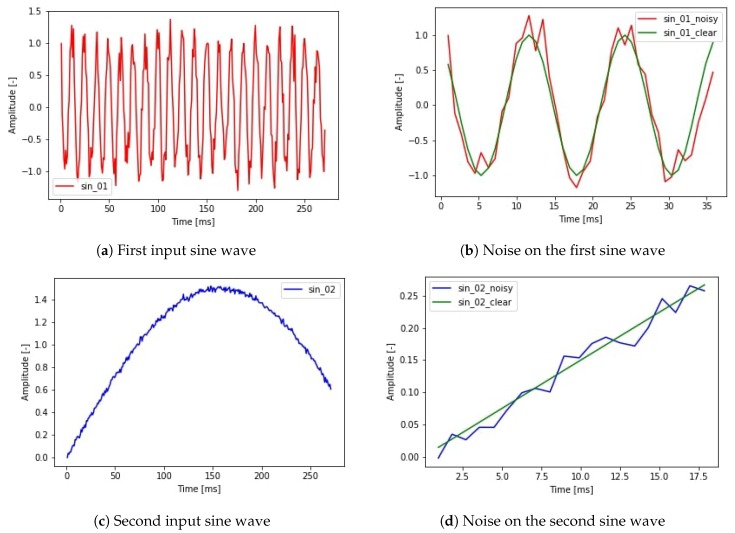
The input features of the simulation: two sine waves with Gaussian random noise.

The simulation can model the noise on the sampling time shown in Figure 10. This noise is present in every application which is not hard real time. Furthermore, noise can be added to the amplitude of each sine function, which creates the output function. The used input signals and the noise on the signals can be seen in Figure 11.

**Figure 12:**
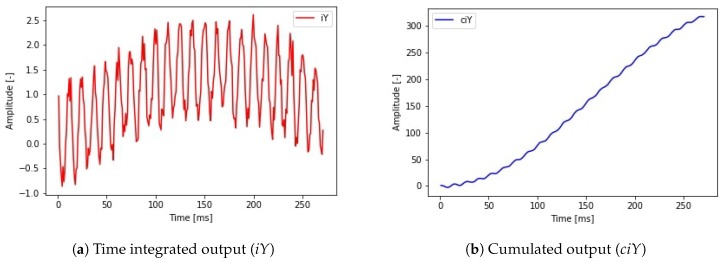
Possible output signals combining the two generated sine waves and sample times with noise.

Combining the two input signals with a desired noise level different output signals are calculated (Equations (9) and (10)). In the first case (Equation (9)) a simple multiplication was implemented. In the second case (Equation (10)) a numerical integral type calculation was made. The difference between the two cases can model the relative position of a robot and the absolute position of a robot. The absolute position calculation task can be defined as summarising the relative position changes in every time step. However, summarising the position information we also summarise the error which creates a shift in the position data. This shift is the result of integrating the noise during the position calculation. This harmful effect is also present in the case of estimating position data from velocity or acceleration data. The possible output signals are depicted in Figure 12. In order to maintain traceability, the epoch number was set to 50. In this case, every teaching task used the same hyperparameters and only the noise level and output signal type changed.

### 4.2. Simulation Results

**Figure 13:**
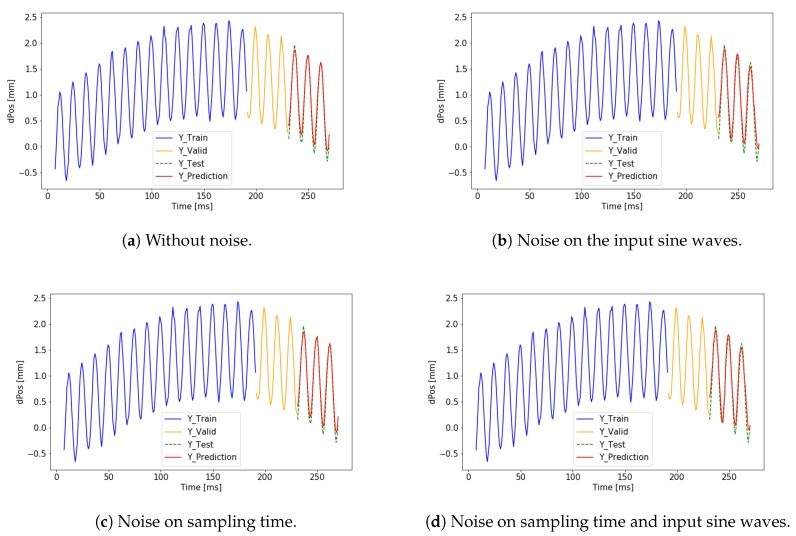
Successful training results on iY without noise on the output.

**Figure 14:**
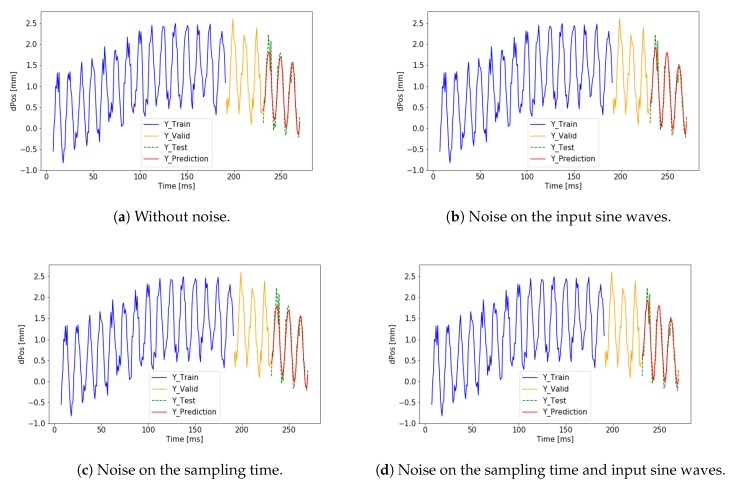
Successful training results on iY with noise on the output.

The simulation environment introduced in Section 4.1 was used to examine different test cases. We focus on the effect of the noise in different learning setups. In the first block, iY, was used as the desired output. The test cases include 3 types of noise. Noise on the sampling time, noise on the amplitude of input sine waves, and noise on the desired output. In every case, the noise was a random noise with Gaussian distribution without offset. The maximum noise level was 5% of the amplitude of the input sine waves. The parameters and the calculated validation loss values are summarized in Table 1. The training data and the visualization of the predicted output can be seen in Figure 13 and Figure 14, where the training data is marked by blue, the validation data set is marked by yellow and the test data set is marked by green, and the prediction is marked by red. The training and validation data sets were used by the algorithm to set the weights and hyperparameters. The test data set was only used to measure the success of the training of the neural network. If the predicted signal (red) overlaps with the reference signal (green) the result of the training is successful. The simulated and the predicted curves are almost identical at the end of the graph and a trend matching can be observed by the different coloring.

**Figure 15:**
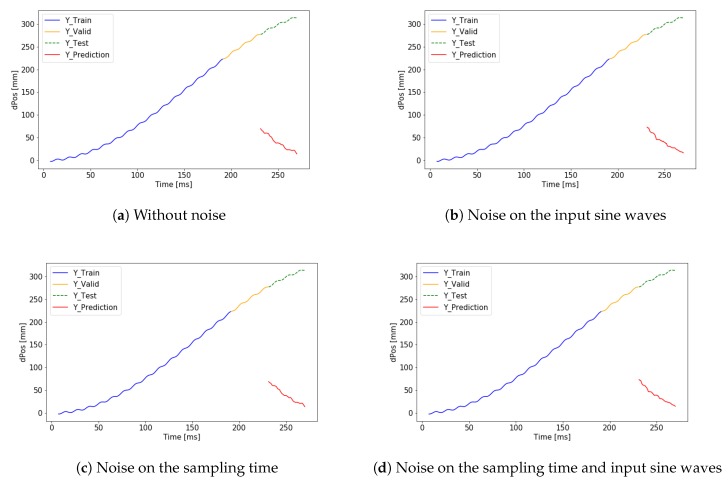
Failed training results on ciY without noise on the output.

**Figure 16:**
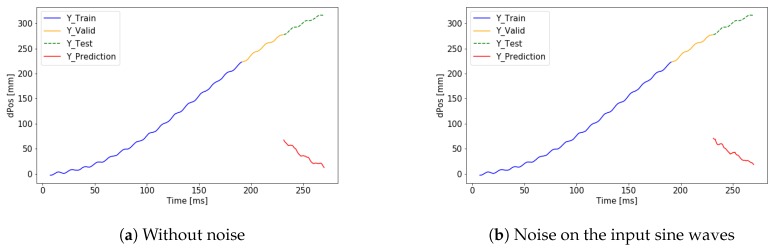

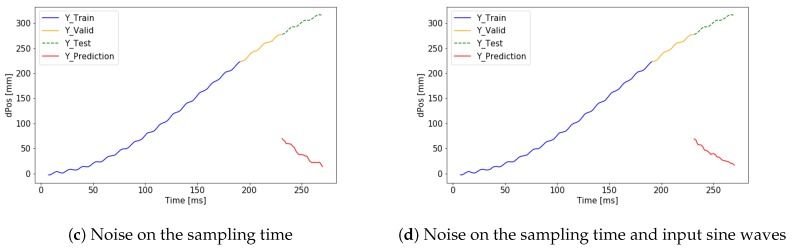
Failed training results on ciY with noise on the output.TnScOnciYTraining

In the second block, ciY was used as the desired output. The numerical test result can also be seen in Table 1. The training data and the visualization of the predicted output can be seen in Figure 15 and Figure 16 where a trend matching failure can be observed. Using the same simple one layer LSTM network the results are better in the first case. The noise level does not cause a significant difference. The change in the output signal, however, caused a big difference. To predict a complex output we need to build a more complex network.

**Figure 17:**
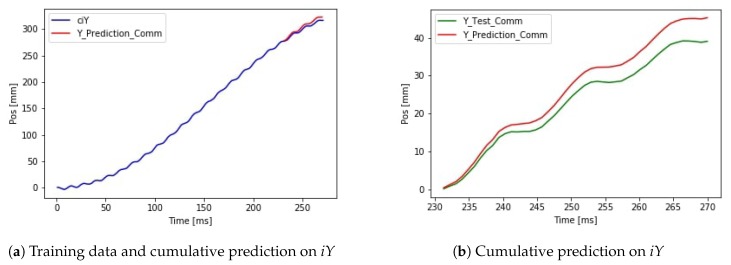
Result of cumulative addition of LSTM prediction on d*z* data.

**Figure 18:**
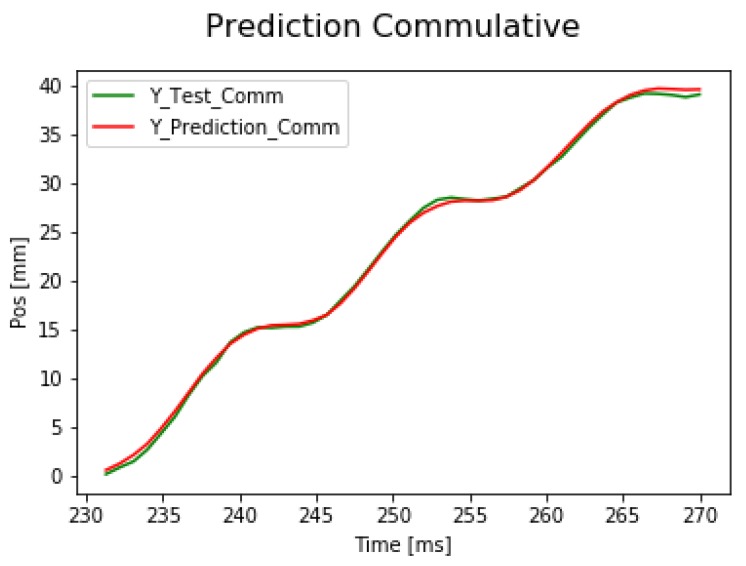
Accurate cumulative prediction by the extended neural network.

First, the signal iY is predicted by the neural network. After summing the predicted small differences, the signal ciY can be calculated. The cumulated error is presented but the validation loss is 14.144 mm, which is significantly smaller than in the case of predicting the absolute position directly. The results can be seen in Figure 17. The performance of the application is improved using a deeper neural network architecture. Using the extended network the validation loss dropped to 2.46 mm. The result of the extended network can be seen in Figure 18.

### 4.3. Simulation Analysis

After running the simulations and checking the results a few guiding ideas were gained before the real-life test. Noise is present in every application. In connection with time serial data based supervised learning task, the noise is also present in the reference data set. In comparison in a supervised categorization task, there is no noise on the labeled reference data during the learning phase. The simulation results show that the maximum 5% noise level on the sampling time, on the input signal amplitude or on the reference signal did not affect the result significantly. The difference between the validation loss values was negligible. The architecture of the neural network and the focus task type caused a significant difference in performance. Because of reduced computational capacity with a small network, the prediction of the small changes in the motion is more accurate than predicting the full trajectory of a movement. The integration task needs a more complex network, however, an acceptable good result can be achieved by a simple network architecture and a numerical integration after the prediction. The prediction error of the extended network on a simulated test case was 14.144 mm.

### 4.4. Measurement Setup

During the real measurements, a Phidget Spatial 3/3/3 sensor [27] was used. This sensor contains a three-axis accelerometer, a three-axis gyroscope, and a three-axis magnetometer. Sampling times are different for the three sensors. The sampling time of the magnetometer is 8 ms, while the sampling time of the accelerometer and gyroscope is 4 ms. The sensor-specific parameters can be seen in Table 2.

Considering future applications during the measurements the sampling time was set to 9 ms. We took some external parameters into consideration like the fact that a 4 ms loop is not enough to take new measurements and calculate the new position estimation on the future robot application. Furthermore, in this case, a not ideal case was tested.

**Figure 19:**
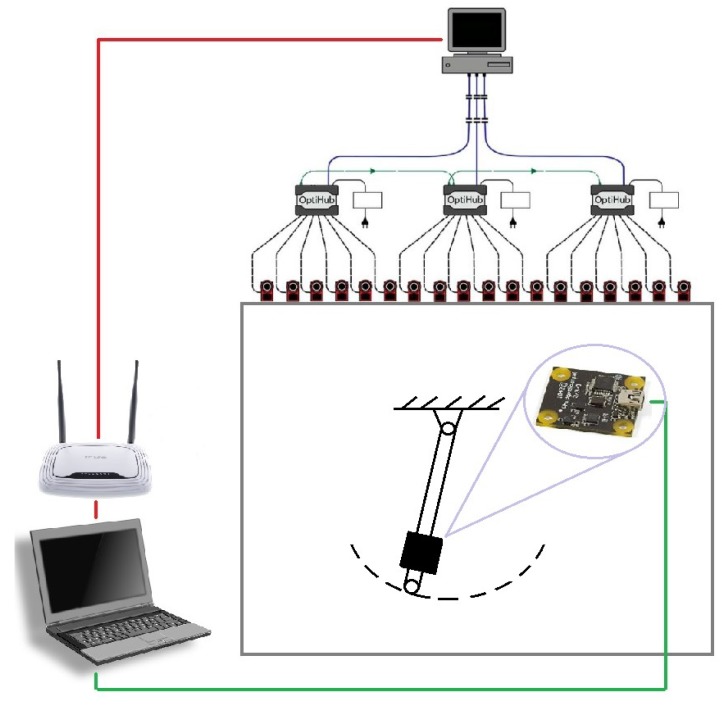
Measurement setup.

The motion capture system update frequency is 120 frames per second (FPS). All of the data was logged on a Lenovo Y520 laptop with an NVIDIA GeForce GTX 1050 graphic card. The laptop collected synchronized data from the sensor and the motion capture system. The schematic drawing of the measurement system can be seen in Figure 19.

**Figure 20:**
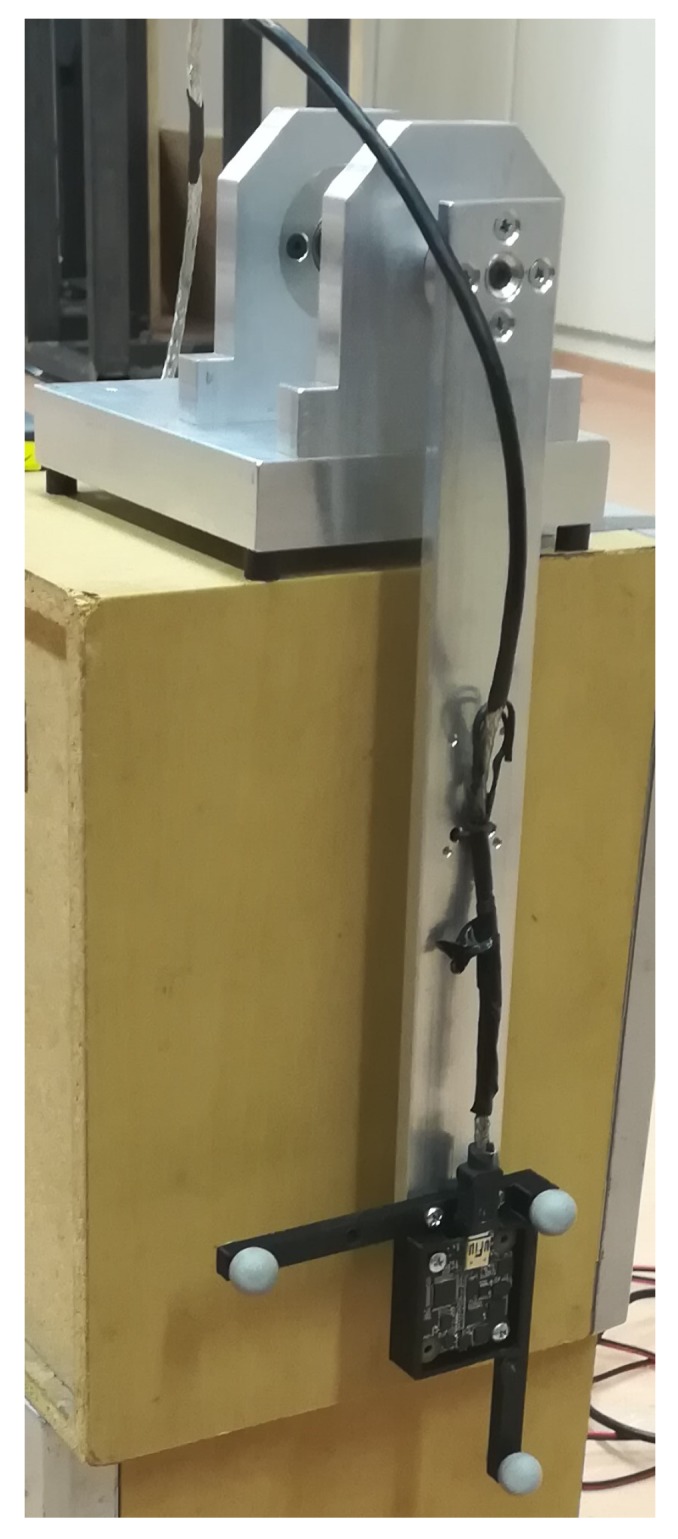
Magnetic, angular rate and gravity sensor (MARG) sensor mounted on a pendulum.

The previously tested and extended neural network model described in Section 3 and shown in Figure 6 was used on a real-life application. The use case is to mount the MARG sensor on a pendulum. The mounted sensor can be seen in Figure 20. The motion capture system can track the three marker as a solid body and provide the position and orientation data for the sensor. During the measurement, the synchronised time, the nine sensor values from the sensor, and the three-position data and four-orientation data in quaternion form from the motion capture system were logged.

### 4.5. Measurement Result

**Figure 21:**
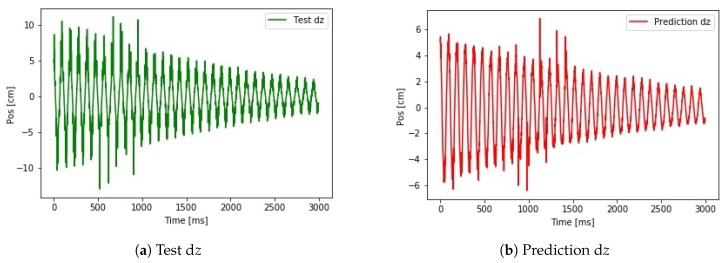

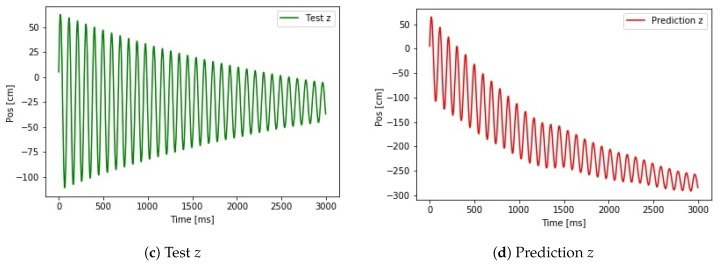
Small LSTM network on real dataset.Test *z*

In the first approximation, the network was tested on the d*z* position values. The training and validation data and the test data are two independent measurements. This prevents any data leak, the algorithm has no information about the test data in the training phase. The reference signals and the predicted signals can be seen in Figure 21.

The shape of the test signal d*z* shown in Figure 21a and the predicted signal d*z* shown in Figure 21b is similar. The test data contains noise, which is not predictable by the network. The error in the max value of the position estimation is due to the difference in the training and test data set. The training data set maximal range of motion was smaller than the maximal range of motion of the test data set. The shallow network was not able to adjust this difference and ended up with a scaling problem. The real problem is the shifting signal after calculating the absolute positions. It means noise with a non-zero offset is appeared in the measurement and integrating the offset the prediction suffered from the shifting.

**Figure 22:**
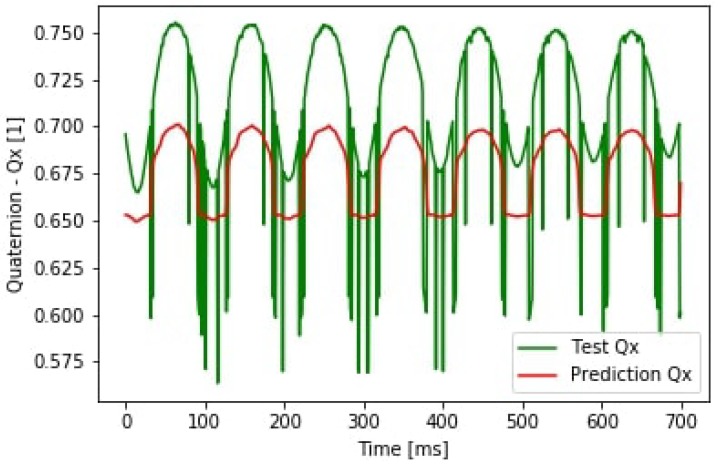
Quaternion Qx prediction.

Examining the orientation output the result of predicting the Qx component of the quaternion vector can be seen in Figure 22. In the figure, an offset prediction is shown. It shows that the reference data from the motion capture system needs additional filtering. This error came from the system itself and causes an offset in the prediction. Compared to the simulation the noise level was much higher during the real test case.

**Figure 23:**
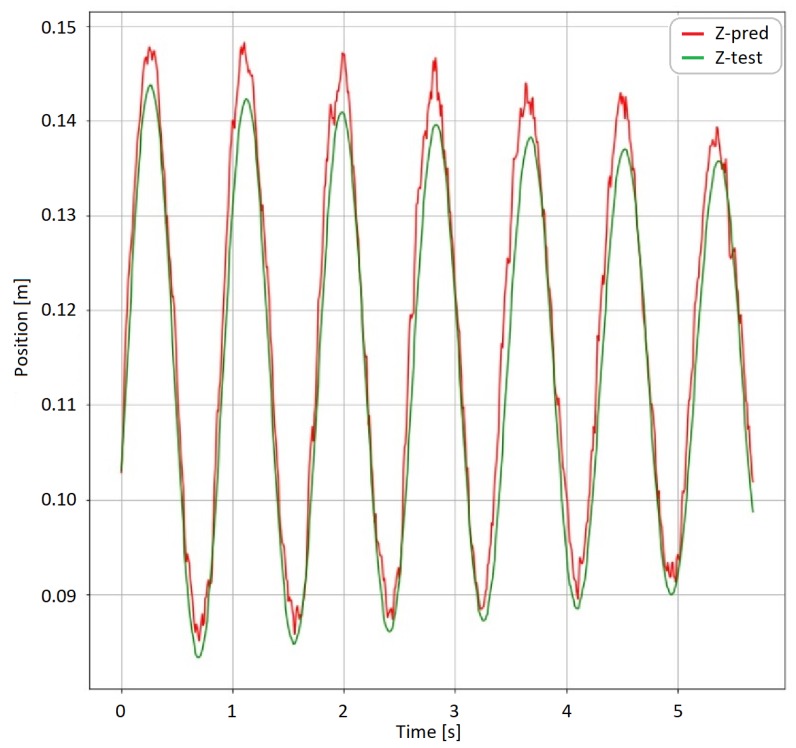
Absolute *z* coordinate prediction.

The neural network model can be modified to learn on the absolute values as well, not only on the position difference data. In this case the shift in the prediction can be eliminated. The result can be seen in Figure 23. The algorithm achieved an average error of 7 mm. The maximum peek error was 43 mm. The shape of the predicted function matches the reference signals. The main reason behind the relative high maximal error is the time delay. The database contained 6324 training samples, 2996 validation samples and 2996 test samples. The test samples were used from a different measurement setup to prevent data leaking during the training phase.

**Figure 24:**
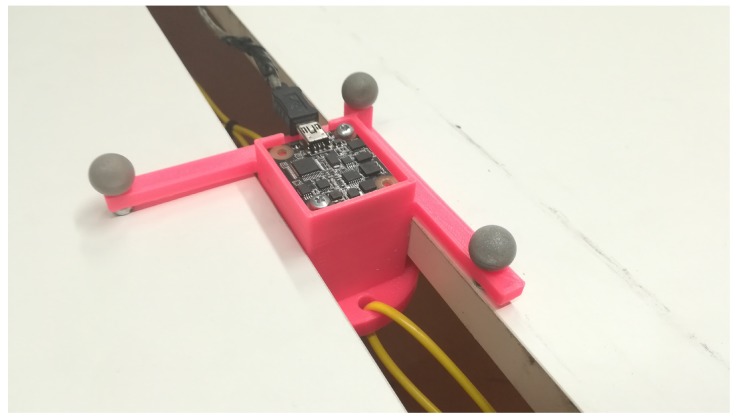
Translational measurement setup.

**Figure 25:**
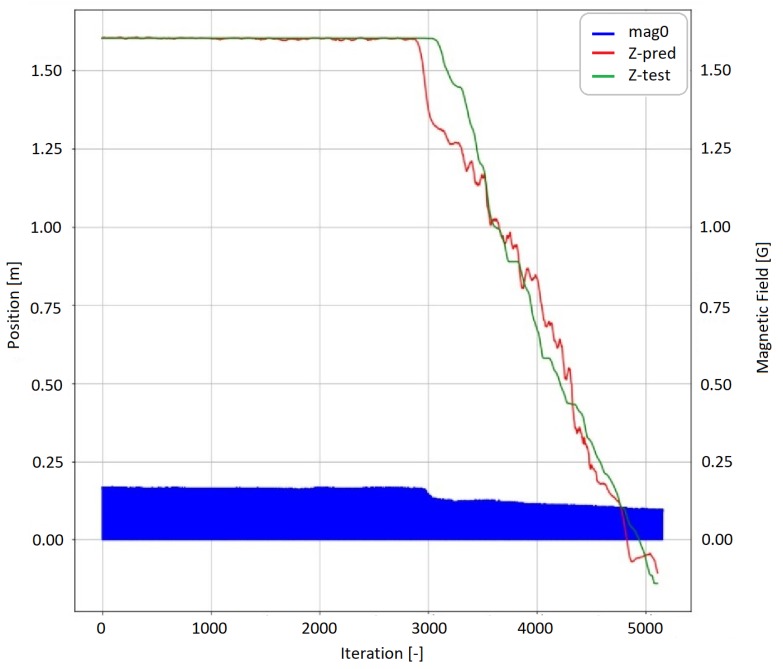
Translational prediction.

As a second measurement setup, the sensor was tested in a translational movement case. The translational measurement test set is shown in Figure 24. In order to test the method in a nonperiodic case, the sensor was moved by a human. The applied force, in this case, was random. The sensor holder was forced to move on a linear path approximately 1.6 m long. The neural network was trained on an additional 30,925 translational measurement points. The iteration time was 0.9 ms. The result of the neural network prediction can be seen in Figure 25. The average error on the test data set is 41 mm, the maximal error is 276 mm. The plot also shows a notable behavior. The neural network output predicted the start of the movement sooner than the reference system detected the movement. Further investigating this particular case the measurement data shows that at the start of the movement the static friction is higher than the kinetic friction. This causes two main changes. Applying the moving force causes a little change in the orientation of the sensor. The magnetometer data, which is also presented in Figure 25 can indicate this event. Furthermore, the sensor holder was moved by a string. Applying the moving force made the string more tense. The stiffness of the string reduced the noise in the accelerometer data providing an extra indicator that a change in the position may occur. To train the neural network on the different cases an NVidia RTX 2080 Ti GPU was used and the implementation was made in Python 3.7. The hyperparameters of the neural network were set up heuristically. In each case, the training phase contained 50 epochs. In the first five epochs root mean square propagation (RMS Prop) [19,20] was the optimization function, while in the next 45 epochs stochastic gradient descent (SGD) [19,20] was used. The batch size was 128. After training the neural network the final neural network architecture was transferred to a system with lower resources. The prediction was made on a i5 520U CPU with 2.2 GHz. The application used 30% of the system resources which is approximately equal to a computational capacity of a Raspberry Pi which is frequently used in low-cost robot and automatization applications. The metrics of the performance and the representative errors can be seen in Table 3.

### 4.6. Discussion

In the simulation the effect of the different noise types was examined in connection with the training process if position estimation can be interpreted as a supervised learning task. A supervised learning process requires a labeled data set however in this case the labeled data set is heavily affected by different noise types. Some relevant cases were investigated in the simulation but not all of them. According to the simulation, the first methodology in Figure 8 seemed better, however, the real-life scenarios are more complex and in this complex case, the second methodology is more suitable.

As a final result of the test of this study the proposed neural network-based approach provides a solution for predicting position from a data of a MARG sensor. Using neural networks on noisy sensor data can reduce the noise, and also can find the hidden structure in the noise. Extracting the information from the changes in the noise structure can improve the prediction ability of an algorithm. The tendency of the prediction shows that the harmful shifting effect of noise integration can be eliminated. Comparing Figure 21d to Figure 23 there is no shift in the prediction. The cumulated error can be reduced by the algorithm using the past position data during the learning phase and the previous prediction data during the test prediction phase.

It was an important criterion to keep computational capacity as low as possible. This implies the training phase as well. This was the main reason to use a not so complex network during the testing. Examining the different effects and behaviors the baseline solution provides valuable information to future development.

This study did not examine the effect of hyperparameter tuning during the development of the neural network. The number of epochs, time steps, input features, and training examples was not optimized during the tests. A neural network performance can be improved by tuning these parameters but this study did not cover these cases. This can be in a focus point of a possible feature study. Experiences from real-life test cases highlighted that the neural network had learned some of the specific features of the measurement setup. Thus the algorithm is not independent from the test case and to make the application more robust this effect can be penalized in a future scenario.

## 5. Conclusions

Using the simulation environment different noise types were examined in periodic case. The simulation result shows that a random Gaussian noise with about 5% of noise level does not affect the learning process fatally. The type of the predicted signal has more effect on the output. Furthermore, according to the simulation it seemed that it is advised to use a smaller, not so complex network with scaled data to predict differences in the signal instead of predicting the complex signals. Finding the optimum point needs more testing and during this study, we did not optimize every hyperparameter. The number of epochs and the number of time steps were constant during the whole testing process. The signal preprocessing is important. The real-life measurements show that the input signals suffer from noise with some offset. Offset noise becomes a shift after an integration. Sensor calibration can be the answer to this problem or can improve the algorithm result. The used Phidget Spatial sensor was calibrated before mounting on the exact measurement setup but it was not enough. In an ideal case, the sensor should be calibrated after the mounting process because the new environment can distort the characteristics of the previously calibrated sensor and likely to cause an offset in the signal level. According to the simulation results it is advised to make a prediction on the differences in the data. In this case, a smaller network can provide relatively good results and the application becomes more robust. However, it works with zero offset noise. If the offset from the noise can not be eliminated or the proper sensor calibration is not possible because of the mounting of the sensor, training on the absolute data is the better choice. The application can handle the offset in the noise both in periodical and non-periodical cases, but the algorithm possibly learns the specialty of the learning case. To show that the proposed technique can work not only for periodic movement a translational movement was measured. The measurement proved that it is a promising approach.

## Figures and Tables

**Table 1:** Summary of different learning cases.

Output Function	Noise on Output	Noise on Input	Noise on Time	Validation Loss (mm)	Figure
iY	No	No	No	0.0077	Figure 13a
iY	No	Yes	No	0.0216	Figure 13b
iY	No	No	Yes	0.0031	Figure 13c
iY	No	Yes	Yes	0.0197	Figure 13d
iY	Yes	No	No	0.0549	Figure 14a
iY	Yes	Yes	No	0.0232	Figure 14b
iY	Yes	No	Yes	0.0555	Figure 14c
iY	Yes	Yes	Yes	0.0241	Figure 14d
ciY	No	No	No	68,670	Figure 15a
ciY	No	Yes	No	66,792	Figure 15b
ciY	No	No	Yes	68,391	Figure 15c
ciY	No	Yes	Yes	68,318	Figure 15d
ciY	Yes	No	No	69,465	Figure 16a
ciY	Yes	Yes	No	69,871	Figure 16b
ciY	Yes	No	Yes	69,509	Figure 16c
ciY	Yes	Yes	Yes	70,024	Figure 16d

**Table 2:** Sensor specific parameters.

Compass	
Compass resolution	400 μG
Offset from North	$2^{\circ }$
**Gyroscope**	
Gyroscope max speed	$400^{\circ }$/s
Gyroscope resolution	$0.02^{\circ }$/s
Gyroscope drift	$4^{\circ }$/min
**Accelerometer**	
Acceleration measurement resolution	228 μg
Acceleration measurement max	±5g
Axis noise (*X*, *Y*)	300 μg
Axis noise (*Z*)	500 μg
**Board**	
Sampling speed max	4 ms/sample

**Table 3:** Summary of different learning cases.

Metric	Periodic	Translational
Samples	6324	30,925
Training time	≈300 s	≈1500 s
Prediction time	4 ms	4 ms
Average error	4 mm	41 mm
Max error	21 mm	276 mm
Range of motion	59 mm	1624 mm
Relative average error	6.7 %	2.64 %

## Supplementary Materials

All the source code of the simulation and different neural networks, and raw measurement data sets used in this paper are available online at https://github.com/Fortuz/MARG-based-supervised-learning. The structure of the project can be found in the README file.
